# A Rule-Based Reasoner for Underwater Robots Using OWL and SWRL

**DOI:** 10.3390/s18103481

**Published:** 2018-10-16

**Authors:** Zhaoyu Zhai, José-Fernán Martínez Ortega, Néstor Lucas Martínez, Pedro Castillejo

**Affiliations:** Departamento de Ingeniería Telemática y Electrónica (DTE), Escuela Técnica Superior de Ingeniería y Sistemas de Telecomunicación (ETSIST), Universidad Politécnica de Madrid (UPM), C/Nikola Tesla, s/n, 28031 Madrid, Spain; jf.martinez@upm.es (J.-F.M.O.); nestor.lucas@upm.es (N.L.M.); pedro.castillejo@upm.es (P.C.)

**Keywords:** underwater robots, semantic representation, ontology languages, SWRL rules, rule-based reasoning

## Abstract

Web Ontology Language (OWL) is designed to represent varied knowledge about things and the relationships of things. It is widely used to express complex models and address information heterogeneity of specific domains, such as underwater environments and robots. With the help of OWL, heterogeneous underwater robots are able to cooperate with each other by exchanging information with the same meaning and robot operators can organize the coordination easier. However, OWL has expressivity limitations on representing general rules, especially the statement “If … Then … Else …”. Fortunately, the Semantic Web Rule Language (SWRL) has strong rule representation capabilities. In this paper, we propose a rule-based reasoner for inferring and providing query services based on OWL and SWRL. SWRL rules are directly inserted into the ontologies by several steps of model transformations instead of using a specific editor. In the verification experiments, the SWRL rules were successfully and efficiently inserted into the OWL-based ontologies, obtaining completely correct query results. This rule-based reasoner is a promising approach to increase the inference capability of ontology-based models and it achieves significant contributions when semantic queries are done.

## 1. Introduction

Underwater environment is characteristically, dynamic and dangerous, leading to several difficulties for human beings to perform underwater operations. For example, it is impossible for human beings to work under high pressure, low temperature, and dark environments for a long time. Under these circumstances, underwater robots [1] are drawing wide attention from many researchers. Underwater robots include heterogeneous robotic vehicles, like Remotely Operated Vehicles (ROVs) [2] and Autonomous Underwater Vehicles (AUVs) [3]. ROVs can be remotely controlled by human operators and AUVs are able to complete assigned missions automatically, such as sea pollution detection [4], underwater infrastructure repairing [5], seabed mapping [6,7], etc. With the help of underwater robots, humans do not need to take any risks by diving into a dangerous underwater environment. Meanwhile, the working efficiency and operation lifetime of underwater robots are much higher than that of human beings.

An underwater robotic system [8] surely contains large quantities of information and data, including communication channels management, underwater environment data, status of robotic vehicles, and mission planning strategies. Substantial problems may happen when heterogeneous underwater robots attempt to exchange disordered information and data. Hence, the underwater robotic system requires certain approaches to model the whole deployment in a well-organized manner, so that the description of the system can be understood both by robotic vehicles and human beings. One of the most popular approaches to model an underwater robotic system is to define the system components and their relationships by ontologies [9,10]. Generally, ontologies encompass a formal representation of certain categories, properties and relations between their concepts, data and entities. Ontologies are widely adopted in many research domains, such as semantic web [11], health care [12], the smart home [13], access control [14], or logistics [15]. An ontology is encoded by means of an ontology language. For example [16]:

- Knowledge Interchange Format (KIF) [17]: It is a formal language for interchanging knowledge between disparate programs and has features like declarative semantics, logic comprehensibility, translatability, and readability. However, KIF requires massive resources to model the whole system.
- The eXtended Markup Language (XML) [18]: It is able to facilitate the representation of task-specific and domain-specific data. XML documents can generate a balanced tree of open and close tags, which contains several attribute-value pairs.
- The Resource Description Framework (RDF) [19]: It can describe any web resources and provide interoperability for exchanging machine-understandable information.
- The Ontology Interchange Language (OIL) [20]: It is a Web-based ontology language that has well-defined semantics with established reasoning properties. One of the advantages using OIL is that it offers various levels of complexity. However, an OIL-based ontology has the difficulties of model transformation. For example, it is not guaranteed that conversions between OIL and RDF can keep the completeness of the model.
- Ontology Web Language (OWL) [21]: As a computational logic-based, OWL is able to represent rich and complex knowledge and it could be exploited by computer programs. OWL is admitted by the World Wide Web Consortium (W3C).

Among the above-mentioned ontology languages, the Smart and Networking Underwater Robots in Cooperation Meshes (SWARMs) project [22], a European project that has its main focus on expanding the use of underwater and surface vehicles to cooperatively perform underwater and offshore operations, adopts the ontology language OWL to model the whole platform. The SWARMs ontology is a Common Information Model and represents several domain-specific knowledge, including communication domain, environment domain, mission planning domain and robotic vehicle domain. The advantage of modeling SWARMs platform with OWL is that OWL is capable for representing the exchanged information between robotic vehicles and enabling robotic vehicles to share and reuse different knowledge. In such networked ontology, it is more convenient for users to search desired resources and deliver the information to computers for further processing.

However, OWL has its own limitations [23,24,25,26,27], such as being unable to represent general rules and statistics-based uncertainty. Generally, OWL can only represent the description logic, leading to weakness of rule-based reasoning. For example, OWL-based ontology is capable of reasoning the correspondence of specific classes, such as $C_{1}\in C_{2}$, meaning that $C_{1}$ set is a subclass of $C_{2}$ set. However, OWL and OWL 2 [28,29] are unable to describe the definition of rule “If … Then … Else”, such as “Elderly”: Person(?x)^hasAge(?x, ?y)^swrlb:greaterThan(?y, 65)→Elderly(?x). This rule indicates that if a person is greater than 65 years old, this person is regarded as elderly. It is worth noting that the representation of general rules plays a significant role in semantics. On the one hand, general rules are able to represent more abundant knowledge, ranging from simple common sense to complex strategies. On the other hand, the representation of general rules can save more memory storage for the knowledge base, because some knowledge could be inferred from existing information by the reasoners. It is unnecessary to store such deducible knowledge in advance. Conclusively, it is quite promising to combine the general rules with OWL-based models.

In 2003, the Semantic Web Rule Language (SWRL) is firstly released as a part of the DARPA Agent Markup Language (DAML) Program [30]. SWRL aims at combining horn-like rules with the OWL-based knowledge base in order to compensate the weakness of OWL [31]. A SWRL rule contains an antecedent (body) and a consequent (head). Both components consist of zero or several atoms. It means that if the conditions specified in the antecedent are reached, then the actions specified in the consequent must be then executed. By adding SWRL rules, the OWL-based ontology can be greatly enhanced in completeness, expressiveness, and logic. This is so because SWRL allows users to specify user-defined rules in order to perform inferences over OWL individuals, so new knowledge about these individuals can be obtained. Moreover, SWRL rules enable the computation of desired behavior based on the content of the individuals by connecting a rule-based reasoner. By updating the new knowledge and rule-based constraints, the OWL-based ontologies become more consistent and may be more applicable to the dynamic environment. For example, the SWARMs users may define two individuals named “VehicleAtLeastSafetyPosition” and “AlarmEvent” and relevant rules to determine the status of robotic vehicles. If one robotic vehicle is at an emergency status while operating the mission, the human operator may be informed by the SWARMs platform and perform immediate operations to recall the vehicles in order to avoid the property loss. The alarm information is generated through the rule-based reasoner based on the current status of the vehicle and user-defined SWRL rules.

Regarding the SWRL rules insertion, some researchers [32,33] recommend to use SWRL plugins of Protégé. Though this approach is easy to use, it can only insert a single rule at one time and the ontology has to be reloaded before the inference process. From the literatures reviews, there are not any published works introducing an approach of inserting multiple SWRL rules at once. In this paper, we propose a novel SWRL rules insertion mechanism in order to directly insert multiple SWRL rules through several steps of model transformations. The proposed rules insertion mechanism does not require users to have the experience or knowledge of editing in Protégé. The users just have to know how to manipulate the SWRL rules and edit them in a text file. The SWARMs ontology is able to adopt the SWRL rules through such text file for further reasoning by the rule-based reasoner.

Additionally, SWRL rules have no limitations on the selections of inference engines, which means that SWRL rules can be reasoned by linking an external inference engine, such as Java Expert System Shell (Jess) [34], Algernon [35], SweetRules [36], and any other user-specified reasoners. This paper proposes a rule-based reasoner using Pellet [37] and Jena [38]. The rule-based reasoner [39,40] is on the basis of “If-Then-Else” statement. The “If” means when the condition is true, the “Then” means take actions, and the “Else” means when the condition is not true, take another actions. Meanwhile, there are two types of rule-based reasoning strategies, including forward-chaining and backward-chaining [41,42,43]. The rule-based reasoner can provide the marine experts and human operators with a comprehensive query services, such as searching for a potential candidate of robotic vehicles for a certain mission, possibility of replacing one robotic vehicle in the swarm, etc.

Conclusively, our main focus is to create and insert the SWRL rules without using any specific editors, like Protégé. Actually, the SWRL rules are manipulated in a text file and then inserted to the SWARMs ontology through several steps of model transformations. Compared with the Protégé, it is worth noting that our proposal allows SWARMs users to insert multiple SWRL rules at the same time instead of inserting the rules one by one. With the successful insertion of SWRL rules, the SWARMs ontology is enhanced with expression capabilities and could provide the SWARMs users with a precise query service by running the rule-based reasoner. The query results generated by the rule-based reasoner are able to help the SWARMs users to take immediate actions in order to make a better decision, obtain higher benefits and avoid economic loss.

The paper is organized as follows. In the next section, the overview of SWARMs ontology and its components are briefly introduced. In Section 3, we describe the construction and syntax of SWRL rules, insertion of SWRL rules into SWARMs ontology. Meanwhile, the rule-based reasoner is represented to provide query services according to inference results. In Section 4, we perform some experiments to evaluate the efficiency of rules insertions and the usefulness of rule-based reasoner. Lastly, we conclude all the contributions and discuss the future work in Section 5.

## 2. Ontologies for Robotic Systems

The term “Ontology” is originated from philosophy, aiming at dealing with realistic objects and conceptual entities. Recently, Artificial Intelligence (AI) and Robotics are adopting this term to represent the real world, enabling the description of objects and the relationships of objects in the knowledge domain [44]. For a robotic system, an ontology is regarded as a formal and clear specification of shared categories, properties, and relations between the concepts, data, and entities. In other words, an ontology of a robotic system captures a common understanding of the domain.

Many researchers tried to build a robotic system by using ontologies. Sadik A.R. and Urban B. [45] combined an ontology-based Multi-Agent System (MAS) and a Business Rule Management System (BRMS) and proposed a distributed control solution to solve challenges in the cooperative manufacturing. It is remarkable that they treated the ontology as a conceptual tool to represent and create a common understanding for the manufacturing work cell entities. Ali F. et al. [46] proposed a merged ontology and Support Vector Machine (SVM) based on an information extraction and recommendation system to convey information and provide recommendations. The ontology-based humanoid robot is able to give accurate feedbacks on a query of a particular topic. IEEE Robotics & Automation Society proposed a Core Ontology for Robotics and Automation (CORA) [47], used by robots that require explicit knowledge. Their ontology is meant to standardize the knowledge representation in the Robotics and Automation (RA) field.

Though plenty of ontologies for robotic systems have been proposed, the construction approaches are not the same. Meanwhile, there is no standard approach of constructing an ontology. Generally, researchers are complying with the following principles to model a robotic system by ontologies [48,49,50]:

1. **Clarity:** An ontology should represent defined terms in a clear and complete manner. The definition of terms should be independent and objective. Definitions could be formularized in logic axioms. Natural languages are recommended to comment on all the definitions.
2. **Coherence:** An ontology should support inferences that are consistent with the definitions. If an inference result contradicts a definition, it means that the ontology is incoherent.
3. **Extendibility:** An ontology should be able to provide the extensions on the existing definitions. An updated definition could be added directly without modifying any existing definitions.
4. **Modularity:** An ontology should have the capability of being divided into several modules with relevant fragments of the ontology. Because a module is usually smaller than the original ontology and is easier to be re-used.
5. **Minimal encoding bias:** The description of terms does not rely on a specific encoding approach. Because the different components of a robotic system in the real world could be programmed by various languages.
6. **Minimal ontological commitment:** An ontology should achieve the minimal ontological commitment, which is exactly sufficient to support knowledge sharing within the ontology. The ontology developers should define as fewer constraints as possible in order to provide freedom to specialize and instantiate the ontology as needed.

### 2.1. Overview of SWARMs OWL-Based Ontology

Based on the above-mentioned principles, the SWARMs project adopts the ontology language OWL to design a networked ontology. The SWARMs ontology [51] has two general characteristics, understandability and conciseness. The characteristics of understandability means that the SWARMs ontology can be easily understood by all stakeholders, including ontology developers, marine experts, human operators, etc. The characteristics of conciseness means that the SWARMs ontology consists of the least number of vocabularies to describe the underwater robotic system.

**Figure 1:**
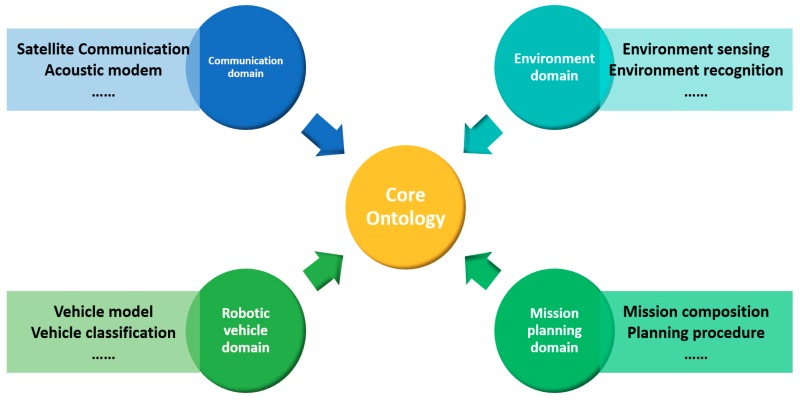
Overview of the ontology in Smart and Networking Underwater Robots in Cooperation Meshes (SWARMs) project.

The SWARMs ontology consists of one core ontology and four domain-specific ontologies, including communication, environment, mission planning, and robotic vehicle domain. The overview of SWAMRs ontology is shown in Figure 1.

**Figure 2:**
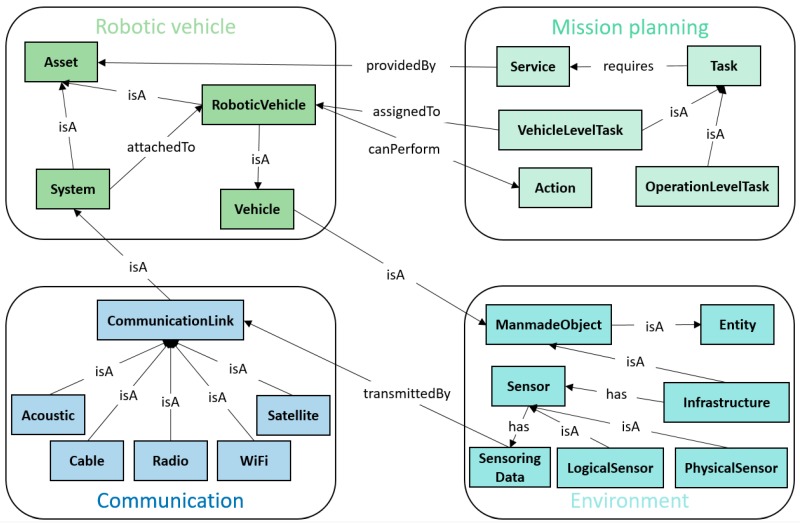
Interconnection of four domain-specific ontologies.

As is shown in Figure 1, the core ontology provides a comprehensive representation and has the capability of interconnecting the four domain-specific ontologies. The interconnection of these ontologies is shown in Figure 2.

The details of four main domain-specific ontologies are explained in the following sub-sections.

**Figure 3:**
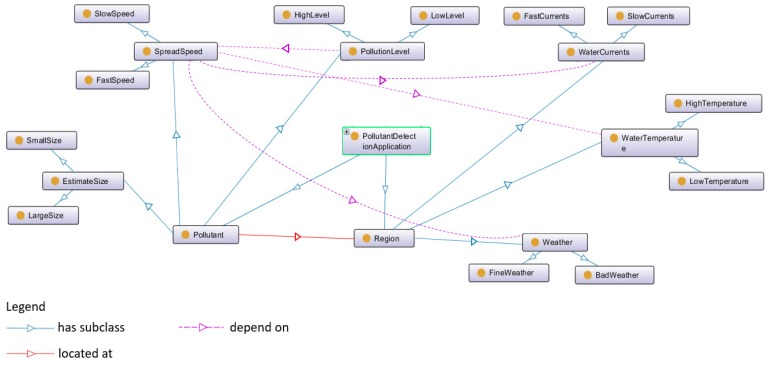
An application ontology of pollution detection.

Besides the four main domain-specific ontologies, application ontologies could be added in order to fulfill specific requirements from different scenarios, such as pollution detection, underwater infrastructure repairing, seabed mapping, etc. The application ontologies aim at enriching the SWARMs ontology and providing the details of assigned missions. For example, an application ontology on pollution detection is given in Figure 3.

With the extension of application ontologies, SWARMs users could perform a pollution detection mission by using underwater robots and surface robots. Based on the environmental variables in the given region, SWARMs users are able to estimate the spread speed of pollutants and conclude the pollution level and estimated size of the polluted area [52].

### 2.2. Communication Domain

The communication domain contains the information model which describes the communication approaches in SWARMs platform. Due to the challenges of unstable communication channels and limited bandwidths, several communication approaches are considered, such as acoustic, cable, radio, satellite, and Wi-Fi. The communication link of acoustic uses an acoustic modem and is able to offer the possibility of wireless communication under water for Autonomous Underwater Vehicles (AUVs) [53]. The communication link of radio and satellite can be used by Remotely Operated Vehicles (ROVs) to transmit data and commands between vehicles and operators [54,55]. If the environment is terrible and the communication is easily disturbed by noise, the communication link of cable is then a reliable option [56]. Meanwhile, several types of messages are defined in the communication ontology, including “ErrorMsg”, “EventMsg”, “NotificationMsg”, “QueryMsg”, “RegistrationMsg”, and “RequestMsg”.

**Figure 4:**
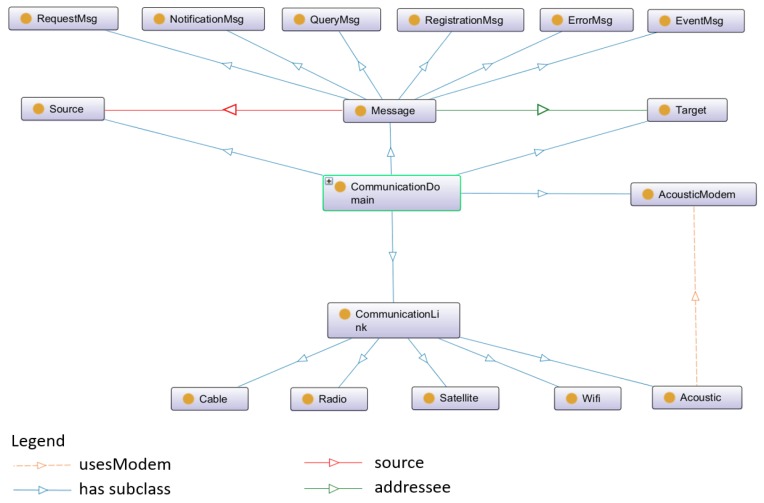
The communication ontology in SWARMs.

The details of communication ontology is defined by OWL in Figure 4.

In Figure 4, the classes of “Source” and “Target” represent the message sender and receiver, respectively.

### 2.3. Environment Domain

**Figure 5:**
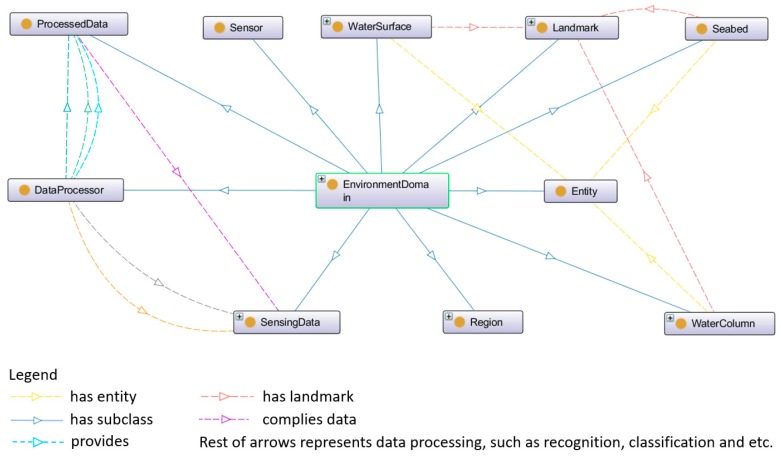
The environment ontology in SWARMs.

The ontology model of environment domain consists of several disjoint classes and describes the concepts within the environment, such as sensors, entities, landmarks, etc. The environment ontology is shown in Figure 5.

**Figure 6:**
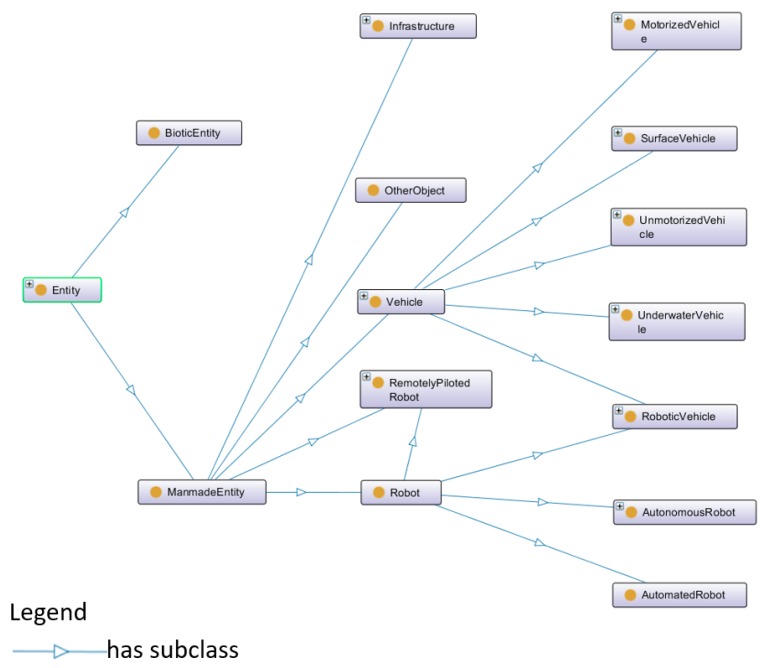
The detail of class “Entity”.

In Figure 5, the class “Sensor” has two sub-classes, named logical sensor and physical sensor. Regarding the physical sensor, it is able to provide physical measurements of the environment, such as water currents and the water temperature. Regarding the logical sensor, it can precept logical data, such as the current condition of a robotic vehicle. The class “DataProcessor” is dealing with the sensing data in order to generate the processed data. For example, “DataProcessor” can perform feature extractions, recognitions and classifications from a captured image. The class “Landmark” refers to specific seabed features, objects, or fixed structures. The class “Entity” represents marine lives and man-made objects. The detail of “Entity” is shown in Figure 6.

### 2.4. Mission Planning Domain

**Figure 7:**
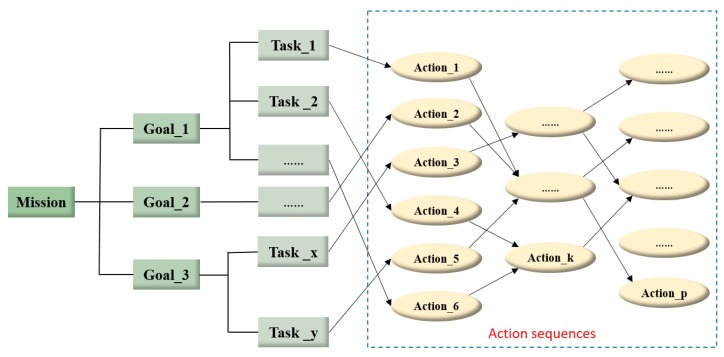
The composition of one mission.

The ontology model of mission planning domain represents the mission composition and planning procedure. Once a mission is established, it is decomposed into several goals according to its objectives and mission specifications. In order to achieve all the goals, certain tasks should be completed by following series of scheduled actions [57]. The details are shown in Figure 7.

**Figure 8:**
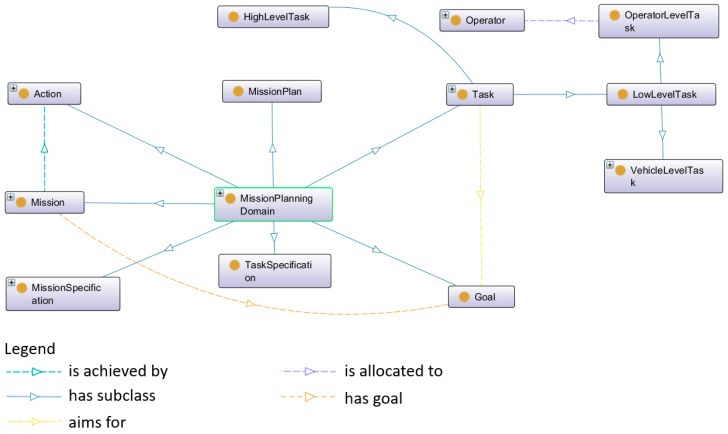
The mission planning ontology in SWARMs.

The ontology model of mission planning domain is shown in Figure 8.

In Figure 8, the class “Task” has two sub-classes, named “LowLevelTask” and “HighLevelTask”. A low-level task can be divided into operator-level task and vehicle-level task. The former one represents that the task requires human operators to manually perform the operations. The latter one means that the task only needs an individual robotic vehicle to carry on the mission, whereas a high-level task has to be executed by a swarm of robotic vehicles.

### 2.5. Robotic Vehicle Domain

**Figure 9:**
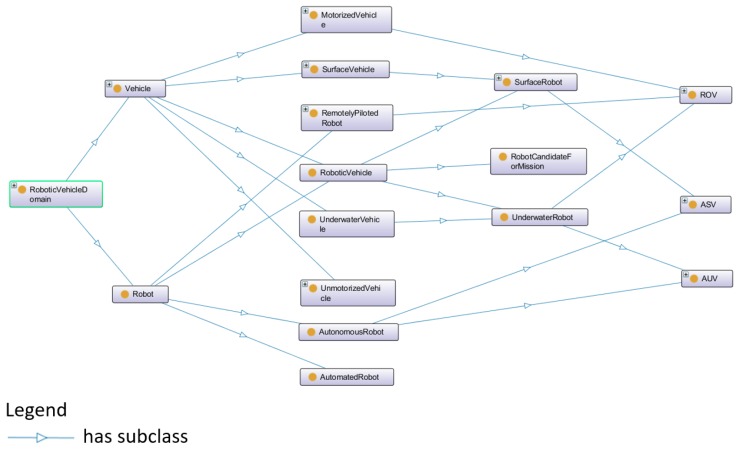
The robotic vehicle ontology in SWARMs.

The ontology model of robotic vehicle domain specifies the robots and vehicles used in the SWARMs project. Generally, a robotic vehicle is either an underwater or surface robot according to its working environment. A robotic vehicle is either an autonomous or non-autonomous according to its autonomy and intelligence [58,59]. The detail of robotic vehicle ontology is shown in Figure 9.

In Figure 9, the class of “RobotCandidateForMission” represents the specific robotic vehicles which have qualified capabilities and fulfil certain requirements of the mission. For example, one certain mission requires that the remaining energy of a selected candidate vehicle should be greater than 60.0 and the vehicle should have certain sensors or actuators. In a robotic vehicle ontology, there are several specific vehicles, including Remotely Operated Vehicle (ROV), Autonomous Surface Vehicle (ASV), Autonomous Underwater Vehicle (AUV), and so on. The detail of these robotic vehicles is as described in [60,61].

## 3. SWRL Rules and Rule-Based Reasoner

As is stated in the previous section, OWL is an ontology description language and has the capability of modelling complex systems and environments. One of the advantages of using OWL is that it has various operators, such as intersection, union, and negation, enabling OWL users to check whether all the definitions and relations in the ontology are consistent. Thus, OWL can represent the SWARMs ontology clearly and succinctly. However, OWL is unable to describe general rules, especially the “if-else-then” statements, leading to impossibility for users to check hidden knowledge in the ontology. Although this knowledge could be defined manually by ontology developers through OWL, this approach definitely increases the complexity of SWAMRs ontology and it actually repeats the existing knowledge. Hence, it is a promising approach to combine the Semantic Web Rule Language (SWRL) to help OWL describing general rules. SWARMs users can define their own rules and insert the rules into the SWARMs ontology for further inferences [62]. With the help of SWRL, the usability of OWL-based ontologies could be extended and the consistency of the ontologies could be improved. The SWARMs users are able to obtain more abundant and precise information by requesting queries to the ontology. A rule-based reasoner can be used to perform inferences on SWARMs ontology with SWRL rules, providing the marine experts and human operators with simple or complex query services, such as searching for a potential candidate among a swarm of robotic vehicles for a certain mission, possibility of replacing one robotic vehicle in the swarm, etc. It is worthy to mention that the proposed rules insertion mechanism allows SWARMs users to directly insert multiple SWRL rules into the SWARMs ontology at one time without editing in Protégé. By this approach, the SWARMs ontology does not need to be reloaded again after the rules insertion.

### 3.1. SWRL Rules

The Semantic Web Rule Language (SWRL) is firstly released in 2003. The latest version of SWRL is updated in 2004 (Version 0.7) with more extensions of Built-Ins. SWRL is on the basis of OWL DL and OWL Lite, enabling the combination of horn-like rules and OWL-based knowledge base. Due to the compatibility of SWRL, OWL can adopt the SWRL rules in order to provide a more completed ontology and enable the possibility of rule-based reasoning.

#### 3.1.1. Structure and Syntax of SWRL Rules

**Figure 10:**
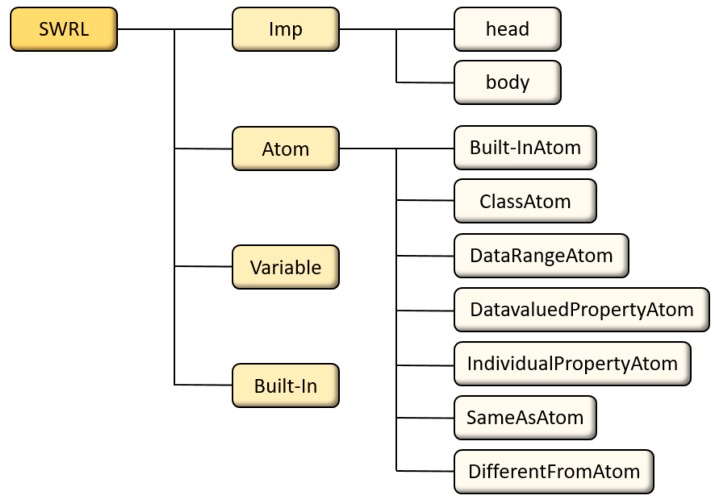
The structure of Semantic Web Rule Language (SWRL) rules.

Generally, SWRL rules consist of four components, including “Imp”, “Atom”, “Variables”, and “Built-in”. The structure of SWRL rules is shown in Figure 10.

In Figure 10, “Imp” is short for implication and consists of head and body. The head and body represent inference results and the initial state before inference, respectively. The basic element in the head and body is “Atom”. The variables of “Atom” are stored in “Variable”. Four basic forms of “Atom” are defined in “Variable” shown as follows.

- C(x). C represents an OWL description or data range.
- P(x, y). P is the property of OWL. x and y can be variables, OWL individuals or OWL data values.
- SameAs(x, y). It represents that x is equal to y.
- DifferentFrom(x, y). It represents that x is different from y.

The “Built-In” is a modular component of SWRL and contains formulas of logic operations, such as boolean operation, mathematical calculation, string operation, etc. Seven types of built-ins are defined in SWRL in Table 1.

As is mentioned before, a SWRL rule consists of an antecedent (body) and a consequent (head). Both antecedent and consequent have multiple atoms. In Listing (1), it describes the abstract syntax of a SWRL rule [63].

(1) rule∷=′Implies('[URIreference]'{annotation} antecedent consequent)'antecedent∷='Antecedent('{atom}')'consequent∷='Consequent('{atom}')'.

In this paper, the SWRL rules are loaded in OWL/RDF format, such as the example given in Listing (2):

(2) $$\begin{array}{l} \langle swrlx:individualPropertyAtom\rangle \\ \;\langle swrl:propertyPredicate\;rdf:resource=“\&\mathrm{eg}:\mathrm{hasBatteryLevel}”/\rangle \\ \;\langle swrl:argument1\;rdf:resource=“\#x1”/\rangle \\ \;\langle swrl:argument2\;rdf:resource=“\#x2”/\rangle \\ \langle swrlx:individualPropertyAtom\rangle \end{array}.$$

The SWRL rules in OWL/RDF format can perfectly merge with the OWL-based SWARMs ontology. Besides, SWRL rules offer a human-readable syntax, such as in Listing (3):

(3) RoboticVehicle(?x)^vehicleCurrentSpeed(?x,?y)^swrlb:equal(?y,0.0)→StandByVehicle(?x).

The above SWRL rule in Listing (3) means that if the current speed of a vehicle is zero, then it is a stand-by vehicle waiting for commands.

#### 3.1.2. SWRL Rules for SWARMs Ontology

With the help of SWRL rules and its human-readable syntax, marine experts and human operators can easily express their knowledge and experience [64] as user-defined rules without knowing the technical details of programming. Usually, pre-defined rules can be embedded in the SWAMRs platform in advance and all robotic vehicles should follow these pre-defined rules to perform underwater operations. However, underwater environment is complex and dynamic. Consequently, marine experts and human operators should add extra new rules and constraints for the purpose of helping robotic vehicles adapting to the environment and carrying on operations safely during runtime. Listing (4)–(9) represent some SWRL rules used in the SWARMs ontology.

(4) RoboticVehicle(?x)^vehicleBatteryLevel(?x,?y)^swrlb:greaterThan(?y,30.0)→RobotCandidateForMission(?x)

In Listing (4), it is a very straightforward and simple rule, representing that if the remaining battery of a robotic vehicle is greater than 30.0, then this vehicle is a candidate for enrolling in the mission. This rule can be specifically inferred by the rule-based reasoner when the SWAMRs platform tries to automatically select the mission candidates. Similar rules of selecting the mission candidates are shown as follows.

(5) RoboticVehicle(?x)^isAvailable(?x,?y)^swrlb:booleanNot(?y,false)→RobotCandidateForMission(?x)

(6) RoboticVehicle(?x)^consumption(?x,?y)^swrlb:lessThan(?y,50.0)→RobotCandidateForMission(?x)

Meanwhile, event-related SWRL rules could be defined by the marine experts. For example, in the mission planning ontology, several types of event is given, including the “AlarmEvent”, “ChangeEvent”, “RequestEvent”, “SignalEvent”, and “TimeEvent”. Marine experts could determine whether a robotic vehicle is at risk or not during runtime by defining a least-safety position. For example, if the remaining battery of a vehicle is lower than a certain value or the position of the vehicle is out of the mission-specified boundary, then it will trigger an alarm event. After being notified, the human operator can recall the robotic vehicle at the least-safety position in order to avoid the property loss. Relevant SWRL rules in SWARMs ontology can be defined as follows.

(7) RoboticVehicle(?x)^GPSPosition(?x,?y)^gpsAltitude(?y,?z)^swrlb:greaterThan(?z,100.0)→VehicleAtLeastSafetyPosition(?x)

(8) RobotCandidateForMission(?x)^vehicleBatteryLevel(?x,?y)^swrlb:lessThan(?y,5.0)→VehicleAtLeastSafetyPosition(?x)

In Listing (7), the SWRL rule represents that if the robotic vehicle detects that its current altitude is greater than 100.0, then this vehicle is at the least-safety position. In Listing (8), the SWRL rule indicates that if the remaining battery of a mission candidate is lower than 5.0, then this vehicle is at the least-safe position. Once the human operator has been notified about the alarm events, immediate actions can be taken to recall these vehicles.

Meanwhile, Marine experts could define the severity level of pollutant by using SWRL rules. The severity level of pollutant depends on its denseness. For example, marine experts could determine the severity level of hydrogen sulfide, also known as H_2_S. The SWRL rule can be defined as follows.

(9) Pollutant(?x)^h2sPollution(?x,?y)^swrlb:greaterThan(?y,20)→SeriousH2SPollution(?x)

In Listing (9), the SWRL rule suggests that if the denseness of H_2_S is greater than 20 parts per million (ppm), then we are dealing with a serious H_2_S pollution problem.

**Figure 11:**
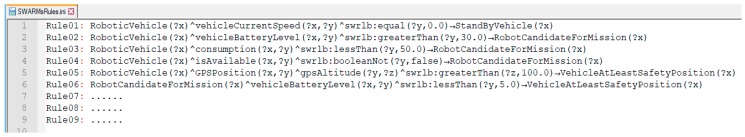
An example of SWRL rule file.

All user-defined SWRL rules are stored in a configuration file under serial orders. An example of such file is given in Figure 11.

Additionally, the rule file can contain only one rule or multiple rules.

### 3.2. Insertion of SWRL Rules into SWARMs Ontology

**Figure 12:**
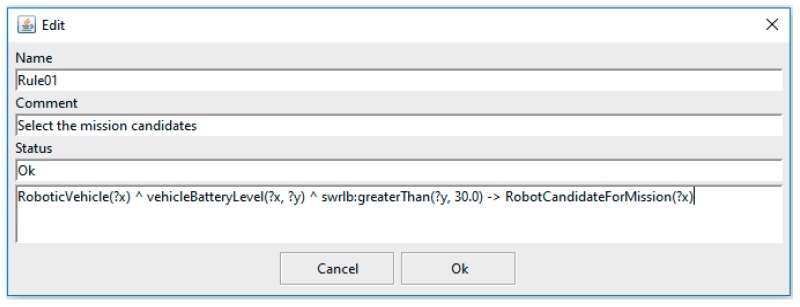
A screenshot of SWRLTab in Protégé.

Most of ontology developers [65,66] are using SWRLTab [67] to insert SWRL rules into OWL-based ontology. SWRLTab is an extension of Protégé-OWL that permits the manipulations on SWRL rules, including creating new SWRL rules, reading and writing existing SWRL rules. A screenshot of SWRLTab is shown in Figure 12.

However, there are some constraints when using the SWRLTab. Firstly, the SWRLTab plugin only allows users to manipulate a single rule at one time. Secondly, it requires the users to have the experience in working with Protégé. Thirdly, it is necessary to have the source code of OWL-based model. Thus, this paper proposes a new approach to insert the SWRL rules into the OWL-based ontology model by several steps of model transformations. The newly proposed rules insertion mechanism allows users to write multiple SWRL rules in a configuration file and then load the rule file into the ontology model. Meanwhile, users just need to understand the SWRL syntax and do not need to use Protégé to edit the SWRL rules. The proposed rules insertion mechanism is one of the novelties in our work.

**Figure 13:**
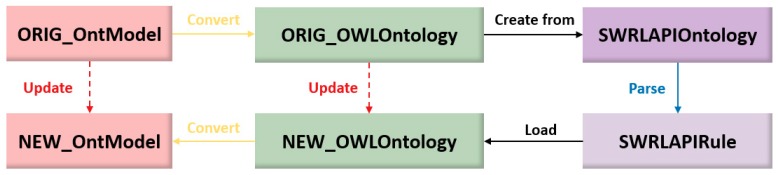
Inserting SWRL rules into Web Ontology Language (OWL)-based ontology model.

The proposed rules insertion mechanism is shown in Figure 13.

In the SWARMs project, the Jena Ontology API [68], OWLAPI [69], and SWRLAPI [70] have been used to manipulate the SWARMs ontology and SWRL rules.

In Figure 13, the steps for model transformations with the purpose of inserting SWRL rules are shown. Normally, the ontology model is fixed and does not allow users to make any modifications, due to the fact that the whole SWARMs ontology is carefully designed and implemented by experts, thus containing highly professional knowledge. The source code of the SWARMs ontology is not fully public and all we receive from the remote server is an ontology file with the “OntModel” (An interface provided by Jena Ontology API) format. However, SWRL rules cannot be directly inserted into the “OntModel”. Therefore, the original ontology model with “OntModel” format has to be converted to the “OWLOntology” (An interface provided by OWLAPI) format. The SWRL rules are parsed by the ontology model with “SWRLAPIOntology” (An interface provided by SWRLAPI) format. Afterwards, a new ontology model with “OWLOntology” format loads all the rules and is converted to “OntModel” format.

**Figure 14:**
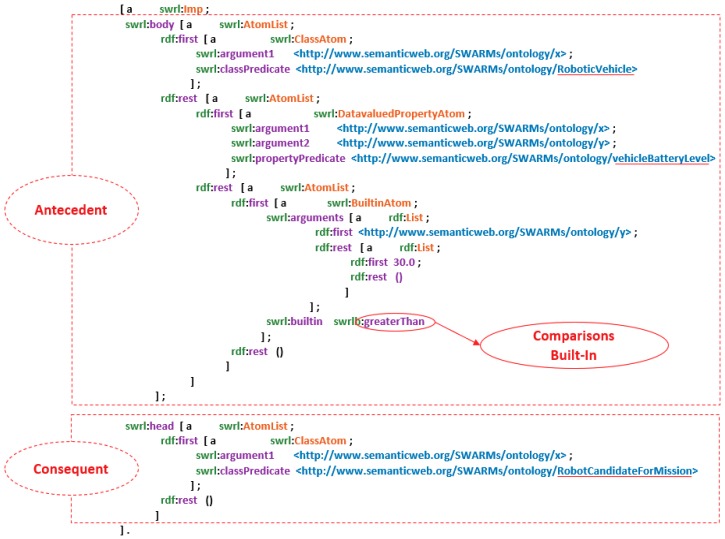
The key part of the rule graph.

According to the specification of “OntModel”, it is an extension to the “InfModel” interface and supports to wrap the abstract syntax of “OntModel” in an RDF graph [71,72]. With the help of this feature, SWRL rules can be wrapped in a RDF graph as well. The rule graph and the ontology graph are independently existed, which means that users may manipulate the SWRL rules without modifying the original SWARMs ontology. Both SWARMs ontology and SWRL rules can be understood in a human-readable manner. The key part of a rule graph is given in Figure 14.

In Figure 14, the atoms of SWRL rules are mapped to RDF triples. The above figure represents the rule Listing (4) in RDF graph. Clearly, the graph consists of two main parts, including the antecedent and consequent. In the body of antecedent, the predicates indicated by the SWRL rule is “RoboticVehicle” and “vehicleBatteryLevel”. Meanwhile, the graph includes a Built-in for comparisons which is “greaterThan”. Thus, the antecedent represents that comparing the battery level of a robotic vehicle, it should be greater than 30.0. In the body of consequent, the predicate is named “RobotCandidateForMission” and it suggests that if the robotic vehicle fulfills the requirement specified in the antecedent, then this vehicle is a potential candidate to execute a mission. Conclusively, the graph represents that if the battery level of a robotic vehicle is greater than 30.0, then this vehicle will be selected as a candidate for one mission.

Generally, after mapping the SWRL rules to RDF triples, the rules are ready to use by the rule-based reasoner in the inference stage.

### 3.3. Rule-Based Reasoner for Inference

**Figure 15:**
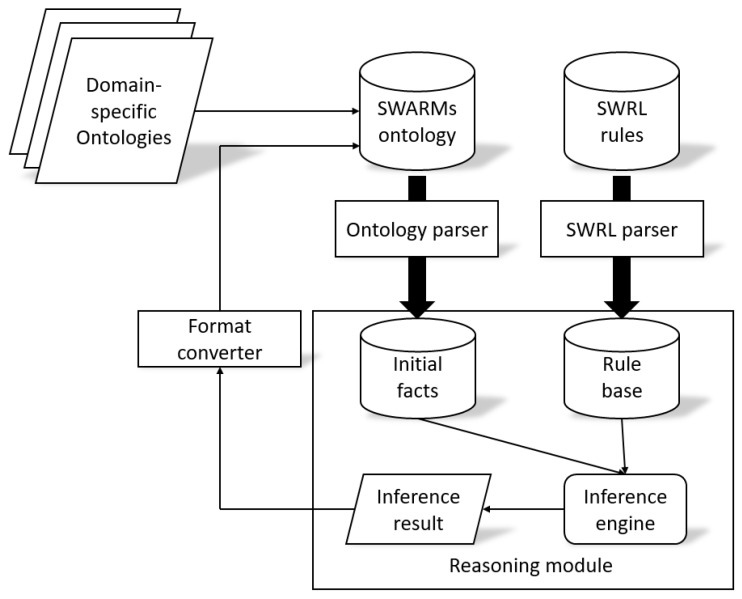
Connecting the SWARMs ontology and rule-based reasoner.

After inserting SWRL rules into the ontology model, the SWARMs ontology is able to connect with a reasoner and generate inference results. This paper proposes a rule-based reasoner to provide SWARMs users with the query services. The rule-based reasoner is a particular type of reasoning approach which uses “If-Then-Else” statements and offers two reasoning strategies, including forward chaining and backward chaining strategies. Generally, the rule-based reasoner is on the basis of initial facts (provided by the SWARMs ontology) and rules (provided by the SWRL rule base). The detail of connection between the rule-based reasoner and SWARMs ontology is shown in Figure 15.

In Figure 15, it is displayed how the knowledge from domain-specific ontologies and SWRL rules are going through corresponding parsers in order to generate initial facts and the rule base respectively. Then, the inference engine loads all the facts and rules and performs rule-based reasoning. The inference results are added as new knowledge into the SWARMs ontology after format conversions.

**Figure 16:**
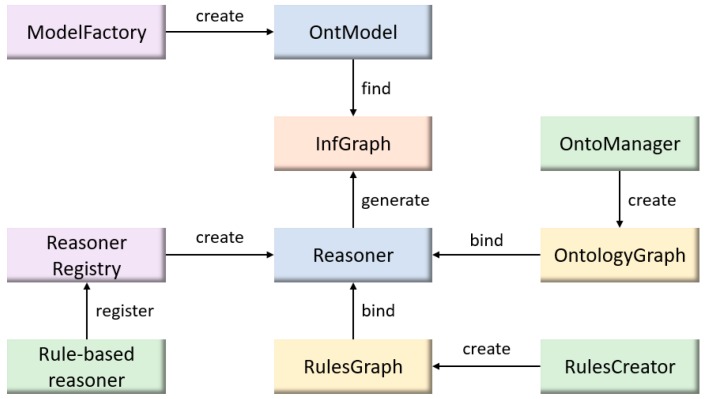
The rule-based reasoner based on Jena inference framework.

The rule-based reasoner is on the basis of Jena inference framework [73]. The details of rule-based reasoner is shown in Figure 16.

In Figure 16, the rule-based reasoner is firstly registered through the reasoner registry module (provided by Jena). Meanwhile, the “ModelFactory” creates a new “OntModel” for the associated reasoner to read knowledge of the ontology. Once the rule-based reasoner is in the reasoner list, ontology graph and rules graph are then loaded in the reasoner for preparation of reasoning. An inference graph is generated and provides the inference results which are reasoned by the rule-based reasoner through forward and backward chaining strategies [74,75].

### 3.4. Query Service

**Figure 17:**
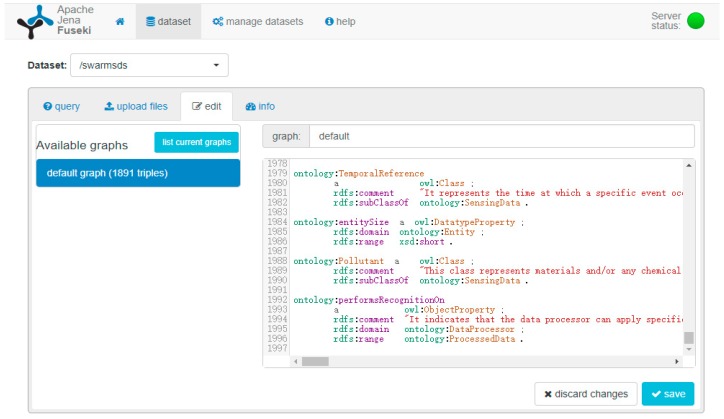
Loaded triples of SWARMs ontology in Apache Jena Fuseki.

The query service is provided for SWARMs users through Apache Jena Fuseki [76]. The Apache Jena Fuseki is basically a SPARQL server and is able to load all the triples of SWARMs ontology in a RDF graph shown in Figure 17.

**Figure 18:**
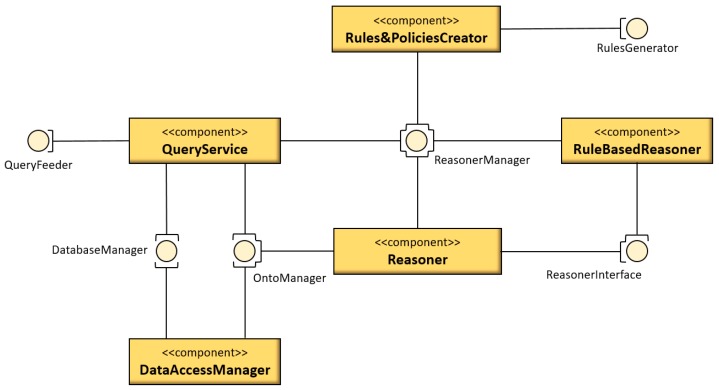
The implementation of query service.

The implementation of the query service is shown in Figure 18.

The query service is implemented by the programming language Java. In Figure 18, the light-yellow round icons represent the interfaces and the dark-yellow rectangle icons represent components (implemented by Java classes). The SWARMs users request the queries through the component “QueryService” and write the SPARQL queries. As is mentioned in Section 3.3, the SWRL rules are created through the component “Rules&PoliciesCreator” and the rule-based reasoner is registered through the interface “ReasonerInterface”. Then, the component “Reasoner” is able to infer the query based on the existing knowledge and user-defined SWRL rules. The query results are lastly returned to the component “QueryService”.

There exists two types of query services, including the simple query and complex query. The simple query refers to the query which does not need any inferences. It is only a query on existing information in the knowledge base. The complex query refers to the query which triggers the rule-based reasoner. It involves the inferring process on the knowledge and may lead to changes on the knowledge base. The detail of these two queries are explained in the following sub-sections.

#### 3.4.1. Simple Query

If the query requested by SWARMs users is just to check existing knowledge in the ontology, then it is unnecessary to trigger the rule-based reasoner for inference. For example, a SWARMs user wants to query on all robotic vehicles which are available for executing certain missions, or the SWARMs user wants to check the remaining battery of certain vehicles. Under this circumstance, this information is already presented in the SWARMs ontology. Thus, the query results can be obtained without using the rule-based reasoner.

#### 3.4.2. Complex Query

Regarding the complex query, it is not a simple query on existing knowledge and it requires the rule-based reasoner for inferring new knowledge. For example, UAV 1 and 2 are involved in a mission and a SWARMs user has doubts on the mission candidates, the user wonders that if UAV 2 can be replaced by UAV 3. Under this circumstance, the rule-based reasoner has to infer the possibility of such replacement based on the status of each UAV and check that whether this substitution is feasible or not. Meanwhile, the SWARMs users may occasionally request a query with plenty of restraints. Some of the restraints may be in conflict with each other, leading to failure of query. Thus, the rule-based reasoner should check that whether the query is correct or not.

## 4. Evaluation and Results

The rule-based reasoner is evaluated by the following aspects. Firstly, the insertion of SWRL rules into the SWARMs ontology is tested. Secondly, the efficiency of rules insertion is recorded. Thirdly, the simple queries and complex queries are requested to verify the correctness of query results. The detail of evaluation is described in the following sub-sections.

### 4.1. Verification of Inserting SWRL Rules

**Figure 19:**
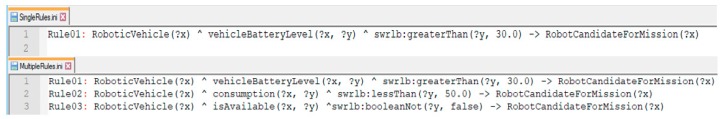
The rule files for testing.

At first, the configuration file (rule file) is given by the SWARMs users. We are testing two rule files, containing a single rule and multiple rules, respectively. The rule files are shown in Figure 19.

**Figure 20:**
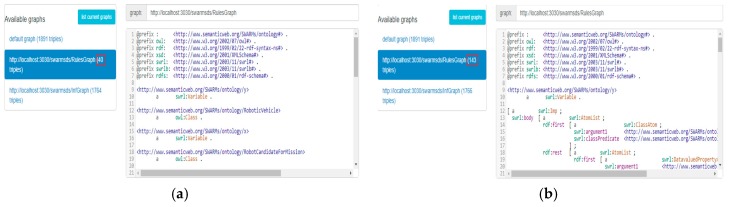
Successful insertion of SWRL rules: (**a**) A single rule; (**b**) Multiple rules.

After uploading the SWARMs ontology to the Apache Jena Fuseki and executing the Java class “PoliciesRulesCreator” (mentioned in Figure 18), the SWRL rules are correctly and fully inserted as a rule graph. The results are shown in Figure 20.

In Figure 20, the number of triples in the rule graph for a single rule is 40 and the number of triples for multiple rules is 143. The content of rule graph is basically the same as the one shown in Figure 14. The results show that the SWRL rules are successfully inserted.

Then, the efficiency of SWRL rules insertion is recorded. In this case, we are testing the rule files which contain 50 rules for three rounds. For each round, a single rule consists of three, four, and five atoms, respectively. The example rules in the rule file for each round are shown in Listing (10)–(12).

(10) pressure(?x)^swrlb:greaterThan(?x,1000)→HighPressure(?x)

(11) RoboticVehicle(?x)^hasVehicleID(?x,?y)^swrlb:startWith(?y,”00”)→ROV(?x)

(12) RoboticVehicle(?x,?y)^UnderwaterRobot(?y,?z)^hasVehicleID(?z,?w)^swrlb:startWith(?w,”00”)→ROV(?x)

**Figure 21:**
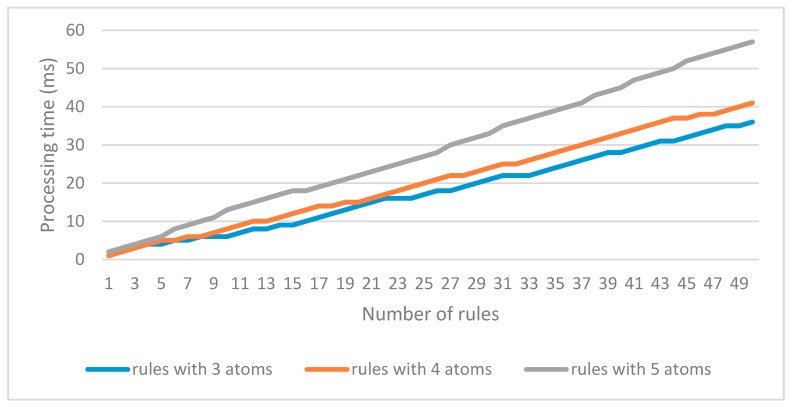
The evaluation of SWRL rules insertion by the proposed approach.

The efficiency of rules insertion is evaluated by the processing time. The result is shown in Figure 21.

In Figure 21, all the SWRL rules are successfully inserted in a very short time. Obviously, more atoms in a single rule, more time is needed. Even a rule consists of five atoms, all the 50 rules can be processed within 60 milliseconds. Compared with inserting SWRL rules by editing in Protégé, the proposed approach is able to insert multiple rules conveniently and efficiently.

### 4.2. Verification of Querying the Udated SWARMs Ontology

**Figure 22:**
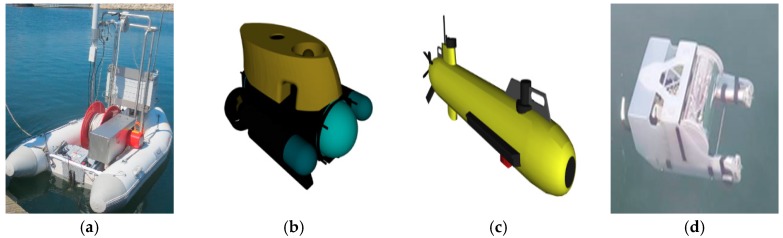
The four instantiated robotic vehicles in the SWARMs ontology: (**a**) SUSV; (**b**) SAGA; (**c**) Alister A9; (**d**) ATN50.

**Figure 23:**
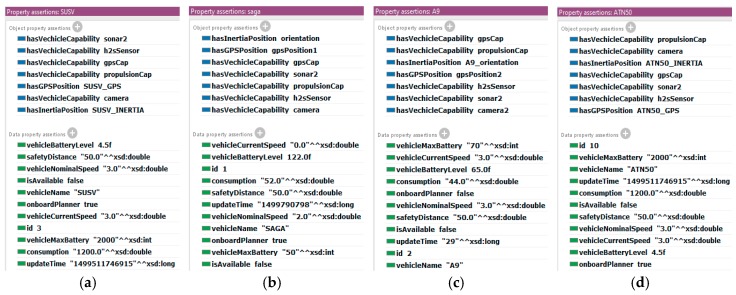
The properties of robotic vehicles: (**a**) SUSV; (**b**) SAGA; (**c**) A9; (**d**) ATN50.

As is mentioned in the previous sections, the rule-based reasoner provides the SWARMs users with query services. In this section, the simple query and complex query are testified respectively. Firstly, in the SWARMs ontology, four instantiated models of robotic vehicles are defined (See Figure 22). Each robotic vehicle model clearly indicates its properties and capabilities (See Figure 23).

**Figure 24:**
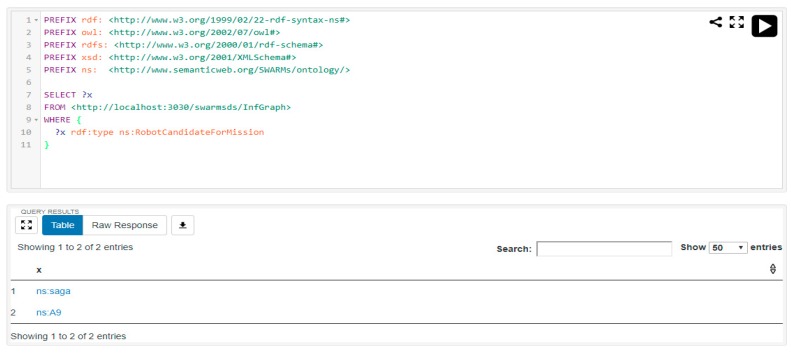
The simple query and result of checking the mission candidates.

Regarding the simple query, it is requested to select the qualified mission candidates. It is assumed that the robotic vehicle with remaining battery power greater than 30.0 will then be selected as a mission candidate. The SPARQL query and the result is shown in Figure 24.

Obviously, in Figure 23, the battery levels of SAGA and A9 are 122.0 and 65.0, respectively. Meanwhile, the battery levels of SUSV and ATN50 are both 4.5. Thus, the robotic vehicles SAGA and A9 fulfill the requirement of being a mission candidate and the query result suggests the same (see Figure 24).

**Figure 25:**
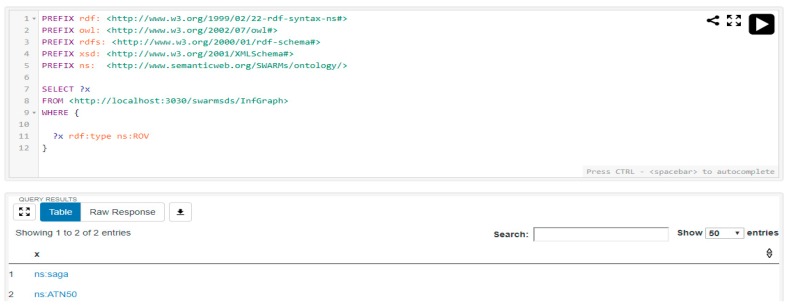
The simple query and result of checking the available ROVs.

Another simple query is also requested by the SWARMs users to check the available ROVs in the SWARMs ontology. According to the specifications of robotic vehicles, SAGA and ATN50 both belong to the class “ROV”. The SPARQL query and the result is shown in Figure 25.

Regarding the complex query, it is requested to check whether a robotic vehicle can return to the base safely or not. The return depends on the current position, speed, and battery remaining of the robotic vehicle and the base position. During runtime, a human operator can request such query for the purpose of property loss. A SWRL rule for determining the safely return is shown in Listing (13).

(13) BaseStation(?bs)^hasGPSPosition(?bs, ?gps2)^RoboticVehicle(?rv)^hasGPSPosition(?rv,?gps1)^vehicleCurrentSpeed(?rv,?speed)^consumption(?rv,?cons)^vehicleBatteryLevel(?rv,?bl)^swrlb:divide(?bl,?cons,)^hasRemainingTime(?remainingTime)^swrlb:subtract(?gps2,?gps1)^hasDistance(?dist)^swrlb:divide(?dist,?speed)^hasReturnTime(?returnTime)^swrlb:lessThan(?returnTime,?remainingTime)→Alert(?alert)^hasRoboticVehicle(?alert,?rv)^hasBaseStation(?bs)

**Figure 26:**
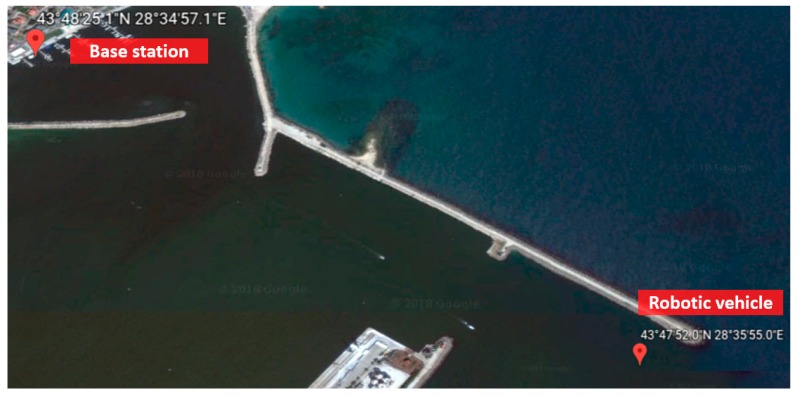
The positions of the base station and robotic vehicle.

**Figure 27:**
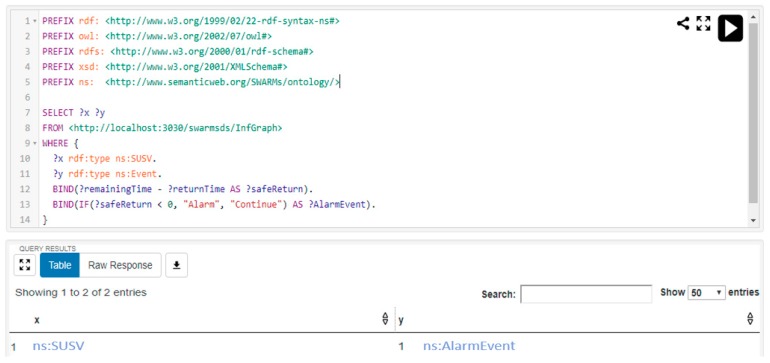
The complex query and result.

The base station is located at 43°48′25.1″ N 28°34′57.1″ E and the current position of the robotic vehicle is located at 43°47′52.0″ N 28°35′55.0″ E (see Figure 26). These two locations are near the Mangalia coast in Romania. It is assumed that the robotic vehicle SUSV is executing a certain mission and the human operator requests the query for the safely return of SUSV. The SPARQL query and result is shown in Figure 27.

In Figure 27, it is depicted that the robotic vehicle SUSV is unable to return back to base safely and corresponding alarm alert is triggered. Obviously, the robotic vehicle SUSV lacks of energy resources according to battery level shown in Figure 23a. Thus, the query result is correct.

Conclusively, the rule-based reasoner is able to perform the reasoning based on the knowledge base and rule base. The inference results can provide the SWARMs users with query services efficiently and intelligently.

## 5. Conclusions and Future Work

The main focus of our work is on the SWRL rules insertions and the query service provided by the rule-based reasoner, along with the detailed implementation. The conclusion of our contribution and future work are summarized in the following sub-sections respectively.

### 5.1. Conclusions

Generally, this paper introduces the OWL-based SWARMs ontology as an information model to enable heterogeneous robotic vehicles to obtain a common understanding of shared knowledge. After analyzing the drawbacks of OWL-based ontology, inserting SWRL rules are proposed as an approach to enhance the expression capability of OWL-based ontology. A rules insertion mechanism is designed specifically for the insertions of multiple SWRL rules. This mechanism allows SWARMs users to efficiently insert multiple rules through a text file at once, instead of inserting the rules one by one in a specific editor. Once all the rules are successfully inserted to the rule base, a rule-based reasoner is able to provide the SWARMs users with query services to know exactly the status of robotic vehicles and desired information of underwater environment. Lastly, experimental tests are performed to verify the proposed rules insertion mechanism and the rule-based reasoner. The experimental results indicate the feasibility and usability of the proposed mechanism and reasoner. Our contribution can be summarized in detail as follows:

- The SWARMs ontology is on the basis of the Web Ontology Language (OWL) and it is capable of representing complex models and address heterogeneous information of the underwater environment and autonomous robots. Therefore, the SWARMs project selects OWL to model the information within the platform. The SWARMs ontology consists of one core ontology and four domain-specific ontologies, including the communication, environment, mission planning, and robotic vehicles domains. As an information model, the SWARMs ontology enables the knowledge sharing and information abstractions in specific domains to help heterogeneous robotic vehicles obtaining a common understanding of shared knowledge. With the help of SWARMs ontology, various robotic vehicles could work with each other for the purpose of completing complex underwater and maritime missions cooperatively. Based on the successful experience of modelling the SWARMs ontology, OWL is a promising approach to represent information of complex environments and robotic vehicles.
- The Semantic Web Rule Language (SWRL) is combined with the SWAMRs ontology for the purpose of overcoming the drawbacks of OWL-based ontologies. OWL-based ontologies cannot represent the general rules, however, with the help of SWRL rules, marine experts and human operators are able to turn their knowledge and experience as user-defined rules and insert them into the SWARMs ontology during runtime. All the SWRL rules are manipulated in a text file before insertion. A rules insertion mechanism is designed and implemented in this paper. The proposed mechanism specifies several steps of model transformations to convert the SWARMs ontology between different formats for the possibility of rule insertions. It is worth noting that the rules insertion mechanism allow SWARMs users to insert multiple rules in one time, which greatly increases the efficiency of rules insertion. Conclusively, SWRL rules could enable the OWL-based ontologies to represent general rules and enhance the completeness of ontologies. Compared with inserting the SWRL rules in a specific editor, the proposed approach does not require the SWARMs users to have knowledge about the editors. Meanwhile, the proposed rules insertion mechanism is more efficient and convenient.
- A rule-based reasoner is implemented to provide the SWARMs users with query services. In the reasoning module, the initial facts of SWARMs ontology and user-defined SWRL rules are loaded to the inference engine from the knowledge base and rule base respectively. Then, the rule-based reasoner is able to perform the inference and generate query results. In this paper, the query service is provided by Apache Jena Fuseki. After all the triples of SWARMs ontology and SWRL rules are loaded in the server, the SWARMs users can write SPARQL queries and request inference results. The query services not only can check the status of robotic vehicles and environment information, but are also able to give feedback to the users regarding the emergency events and decision corrections. It is concluded that the rule-based reasoner is a promising approach to increase the inference capability of SWARMs ontology and provide the SWARMs users with precise query services.
- Lastly, experimental tests are performed in order to verify the proposed rules insertion mechanism and the rule-based reasoner. In this paper, the success of rules insertion and the insertion efficiency are tested. No matter inserting a single rule or multiple rules at once, the successful insertions are ensured with high efficiency. Afterwards, the rule-based reasoner is testified by requesting simple and complex queries. The query results are totally correct. Consequently, the rules insertion mechanism and rule-based reasoner are feasible and useful for the SWARMs project.

### 5.2. Future Work

Though the SWRL rules are able to enhance the expression capability of OWL-based ontologies and the rule-based reasoner is a promising approach for providing the SWARMs users with a convenient query services, further developments are still pending to be implemented for the purpose of improving the SWARMs ontology, SWRL rules, and rule-based reasoner. The future work is summarized as follows:

- Firstly, regarding OWL, it is necessary to extend and enrich the SWARMs ontology with more elements, such as various robotic vehicles, communication approaches, user cases, and environment landmarks. Meanwhile, though the SWARMs ontology is designed to provide a common information model for underwater environment and robotic vehicles, the reusability of such ontology should be taken into consideration for other underwater robotics projects.
- Secondly, it is worth exploring the extensibility of SWRL rules. Apart from the expressions of general rules, statistics-based uncertainty also existed in the SWARMs project. It is promising to implement a probabilistic extension of SWRL to deal with the incomplete or partial knowledge. With the help of probabilistic extension, certain reasoning algorithms (such as Multi-Entity Bayesian Network algorithm) could be executed in order to infer uncertainties, providing an improved query services to the SWARMs users.
- Thirdly, a conflict-free mechanism for rules insertion should be developed in order to avoid the redundancy and complexity of the rule base. Repeated rules and conflict rules may result in difficulties for the rule-based reasoner to understand and perform inferences. The conflict-free mechanism aims at filtering the SWRL rules and eliminating unnecessary ones, keeping the simplicity of the rule base in this way.

## Figures and Tables

**Table 1:** The Built-Ins in SWRL.

Built-In	Example	Example Explanation
Comparisons	swrlb:equal	If the first argument and the second argument are the same.
Math	swrlb:abs	If the first argument is the absolute value of the second argument.
Boolean Values	swrlb:booleanNot	If the first argument is true and the second argument is false, or vice versa.
Strings	swrlb:startsWith	If the first argument starts with the second argument.
Date, Time and Duration	swrlb:time	If the first argument is the xsd:time representation, followed by arguments of hours, minutes, seconds and timezone.
URIs	swrlb:anyURI	If the first argument is a URI reference, followed by arguments of scheme, host, port, path, query and fragment.
Lists	swrlb:empty	If the first argument list is empty.
